# Efficacy and Safety of Subcutaneous Infusion of Non-formulated Furosemide in Patients with Worsening Heart Failure: a Real-World Study

**DOI:** 10.1007/s12265-021-10173-1

**Published:** 2021-10-12

**Authors:** Jose Civera, Rafael de la Espriella, Raquel Heredia, Gema Miñana, Enrique Santas, Adriana Conesa, Anna Mollar, Clara Sastre, Ana Martínez, Amparo Villaescusa, Julio Núñez

**Affiliations:** 1 Cardiology Department, Hospital Clínico Universitario de Valencia, INCLIVA, Av. Blasco Ibáñez 17, 46010 Valencia, Spain; 2 Departamento de Medicina, Universitat de València, Valencia, Spain; 3 CIBER in Cardiovascular Diseases (CIBERCV), Madrid, Spain

**Keywords:** Worsening heart failure, Subcutaneous furosemide, Urinary sodium, Diuretics, Fluid overload

## Abstract

We aimed to evaluate the efficacy (short-term changes in surrogates of decongestion) and safety following the ambulatory administration of subcutaneous furosemide (SCF) in patients with WHF. Fifty-five ambulatory patients were treated with SCF administered by an elastomeric pump for at least 72 h. Surrogates of congestion were assessed at baseline, 72 h, and 30 days. Spot urinary sodium (uNa+) was assessed at baseline, 24-48-72 h, and 30 days. The median (IQI) of NT-proBNP and uNa+ at baseline was 5218 pg/mL (2856-10878) and 68±3 mmol/L, respectively. Following administration of SCF (median dose of 100 mg/daily), we found a sustained increase in uNa+ during the first 72 h of treatment compared to baseline, paralleled with evidence of decongestion at 72 h, and 30 days. No significant safety concerns were observed. SCF was an effective and safe diuretic strategy for outpatient congestion management.

**Graphical abstract:**

**Figure:**
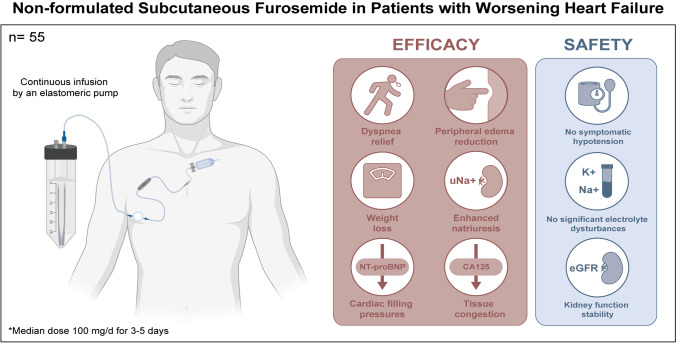
Non-formulated subcutaneous furosemide in patients with WHF. Efficacy and safety.

**Supplementary Information:**

The online version contains supplementary material available at 10.1007/s12265-021-10173-1.

## Introduction

Fluid overload is the leading cause of morbidity in heart failure (HF) patients and is responsible for most decompensations [1]. Traditional therapeutic approaches for managing these patients include hospital admission and administration of intravenous loop diuretics [1, 2]. Alternative diuretic strategies to successfully and safely treat most HF decompensations in an ambulatory setting (*Hospital at Home*) remain non-well validated. Some studies reported promising efficacy and safety results of home-based diuretic therapeutic strategies for managing patients with worsening heart failure (WHF) [3, 4]. In most cases, they include increasing the dose of oral diuretics and intermittent administration of intravenous or subcutaneous loop diuretics [3–5]. However, the efficacy of subcutaneous furosemide (SCF) in daily clinical practice is scarce and mostly limited to palliative care settings [3, 4]. Most of the evidence suggests the relief of symptoms, but there is not robust information about other parameters of fluid overload and response to diuretic therapy, such as changes in urinary sodium (uNa+), natriuretic peptides, and antigen carbohydrate 125 (CA125); all of them are reliable tools for monitoring diuretic response in patients with WHF receiving intravenous loop diuretics [6–9].

This study aimed to evaluate the short-term changes in surrogates of decongestion/response to therapy [uNa+, New York Heart Association (NYHA) class, dyspnea visual analog scale (VAS), pedal edema score, weight, plasma amino-terminal pro-brain natriuretic peptide (NT-proBNP), and antigen carbohydrate 125 (CA125)] after treatment with SCF for at least 72 h in patients with WHF and volume overload. Additionally, safety parameters in terms of short-term changes in eGFR, serum sodium (Na+) and potassium (K+), and systolic blood pressure (SBP) were also assessed.

## Methods

### Study Design and Patients

Patients included in this cohort received an SCF infusion for the treatment of WHF between Nov 7, 2019, and Jan 1, 2021, at an outpatient HF-Clinic in Spain (Hospital Clinic Universitari, Valencia-Spain). Patients were eligible if they presented with WHF and fulfilled at least one of the following inclusion criteria: (1) objective evidence of structural or functional cardiac abnormality documented either by systolic left ventricular dysfunction (left ventricular ejection fraction <50%), left ventricle hypertrophy (defined as a septum or left ventricular posterior wall thickness ≥12 mm, or left ventricular mass index >104 g/m^2^ in women or 116 g/m^2^ in men), *E*/*e*′ ratio >15, or significant valvular heart disease (moderate to severe); (2) NT-proBNP >1000 pg/mL; (3) history of symptomatic chronic HF on active treatment with oral loop diuretics (i.e., furosemide or torsemide). Exclusion criteria consisted of (1) acute decompensated heart failure requiring hospital admission (acute pulmonary edema, evidence of hypoxemia defined as an oxygen saturation <90% in pulse oximetry or oxygen partial pressure <80 mmHg in arterial blood gas analysis), (2) cardiogenic shock, (3) symptomatic hypotension or any SBP <90 mmHg, (4) index event triggered by an uncontrolled arrhythmia (advanced heart block without a pacemaker, sustained ventricular tachycardia, therapeutic defibrillator shock, or atrial fibrillation/flutter with sustained ventricular response >150 beats per minute), infection/sepsis, or severe anemia (hemoglobin < 7 g/dl). Patients on renal replacement therapy or ultrafiltration were also excluded. This study complied with the Declaration of Helsinki and was approved by the local institutional review committees.

### Study Intervention

SCF was administered by using a single-use, continuous infusion pump system (DOSI-FUSER®, Leventon, S.A.U, Barcelona, Spain) and a standard commercial subcutaneous infusion set. The infusion pump system consists of an elastomeric balloon inside a rigid container, an infusion line with the capillary device, and a Luer-lock connector that attaches to the standard subcutaneous infusion set. After the balloon is inflated, the medication flows through the capillary device as a result of the pressure from the elastomeric balloon, which determines the flow rate. For the present study, we used an infusion pump containing a 250 mL balloon reservoir with a nominal continuous flow rate of 2.1 ml/h over a median of 72 (72–72) hours.

SCF dose was calculated based on the subject’s outpatient oral dose using a 1:1.25 conversion (80 mg of oral furosemide = 100 mg of SCF). Therefore, for administering a daily dose of 100 mg of SCF, a 2mg/mL drug concentration was required (dilution: 500 mg of non-formulated furosemide in 250 mL of 0.9% sodium chloride). Specialized HF nurses filled the infusion system following the manufacturer’s instructions, placed the subcutaneous catheter, and thoroughly explained general guidelines and troubleshooting to study participants.

### Clinical Monitoring and Endpoint Assessment

All patients were physically visited at baseline, 72 h, and 30day. At these encounters, we registered the NYHA class, dyspnea VAS, pedal edema grading scale, weight, vital signs, 12-leads electrocardiogram, and standard plasma laboratory data (including estimated glomerular filtration rate (eGFR), plasma electrolytes (sodium and potassium), NT-proBNP, and CA125). All additional visits were also documented. The dyspnea VAS scale of 0 corresponds to the patient’s subjective feeling of “I Can Breathe Normally,” and a dyspnea VAS score of 10 corresponds to “I Can’t Breathe At All.” The pedal edema was assessed on a standardized 4-point scale ranging from 0 to 3 (0, absent/trace; 1, slight; 2, moderate; 3, marked). The 72-h cumulative volume of diuresis was registered in only 38 patients.

### uNa+ Assessment

During the baseline visit, patients received a thorough explanation of how to collect ambulatory uNa+ samples. Briefly, patients were instructed to collect a daily first void morning urine sample. Patients taking other diuretics in the morning were guided to take the diuretic only after collecting the first-morning void. Urine was collected in a disposable urine collection cup. Afterward, a spot sample was aspirated from the sealed collection cup by using the aspiration port. The patients were told to immediately place the labeled vacuum tube containing the urine in their freezer (with a commercial standard of approximately −18°C). On the same morning, patients registered data on their 24-h diuresis. uNa+ was registered each 24-h after treatment intervention at 24, 48, and 72 h. After removing the infusion pump system, uNa+ was also measured at each additional physical encounter and 30 days. Patients were instructed to collect and refrigerated the home urine sample at 24 and 48 h.

### Objectives

The study’s efficacy endpoint was to determine the changes in short-term (72 h and 30 days) surrogates of congestion (NYHA class, dyspnea VAS, pedal edema grading scale, weight, NT-proBNP, and CA125) and diuretic response assessed by uNa+ at 24, 48, 72 h, and at 30 days, compared to the baseline value. Additionally, short-term changes in eGFR, serum sodium (Na^+^) and potassium (K^+^), and systolic blood pressure (SBP) were also assessed as safety endpoints.

### Statistical Analysis

As appropriate, continuous baseline variables were expressed as mean ± standard deviation or median [interquartile interval (IQI)]. Discrete variables were presented as numbers (percentages). Changes in continuous endpoints and their longitudinal trajectories were estimated with linear mixed regression models (LMRMs). Continuous exposures with a non-parametric distribution were log-transformed [NT-proBNP (lnNT-proBNP) and CA125 (lnCA125)]. Unadjusted and adjusted estimates were presented. Multivariate estimates were adjusted for age, sex, baseline eGFR, left ventricular ejection fraction (LVEF), and the baseline endpoint value regardless of their *p* value. The LMRMs are presented as least square means (LSM) with their respective 95% confidence intervals. *p* values were adjusted for multiple comparisons (Sidak procedure). A 2-sided *p* value of <0.05 was set as a criterion for statistical significance. All analyses were performed in Stata 15.1 (Stata Statistical Software, Release 15 [2017]; StataCorp LP, College Station, TX, USA).

## Results

The mean (SD) age of the sample was 79 ± 8 years, 23 (41.8%) were women, 53 (96.4%) patients showed NYHA class III at baseline, and 26 (47.3%) showed LVEF >50%. All study participants (100%) were previously treated with oral loop diuretics, and 39 (70.9%) were receiving more than two diuretics at the moment of inclusion. The median [interquartile range (IQR)] dose of oral furosemide and SCF was 80 (80 – 80) and 100 (100 – 100) mg/daily, respectively. At baseline, the mean VAS, systolic blood pressure, eGFR, and uNa+ were 7.4 ± 1.4, 122 ± 20 mmHg, 46 ± 20 mL/min/1.73m^2^, and 68 ± 3 mmol/L, respectively. The median (IQR) of NT-proBNP and CA125 was 5218 pg/mL (2856–10878) and 54 U/mL (22–138), respectively. Detailed baseline characteristics of the sample are presented in Table 1. In those in which cumulative diuresis was registered, the median diuresis volume during the first 72 h was 6000 mL (5000–8500).

**Table 1:** Baseline characteristics

Variables	Study population (*n*=55)
*Demographics, medical history, and vital sings*	
Age, years	79.0 ± 8.0
Male, *n* (%)	32 (58.2)
Hypertension, *n* (%)	48 (87.3)
NYHA, *n* (%)	
II	2 (3.6)
III	53 (96.4)
Diabetes mellitus, *n* (%)	25 (45.5)
COPD, *n* (%)	4 (7.3)
Dyslipidemia, *n* (%)	45 (81.8)
Ischemic etiology, *n* (%)	8 (14.5)
Valvular disease, *n* (%)	6 (10.9)
Renal failure, *n* (%)	36 (73.5)
Atrial fibrillation, *n* (%)	42 (76.4)
Pedal edema grading scale, *n* (%)	
0 (absent/trace)	1 (1.8)
1 (slight)	26 (47.3)
2 (moderate)	11 (20)
3 (marked)	17 (30.9)
Pleural effusion, *n* (%)	10 (23.8)
Jugular engorgement, *n* (%)	33 (60)
VAS	7.4 ± 1.4
Heart rate, bpm*	70 (63–86)
SBP, mmHg	122.5 ± 19.5
DBP, mmHg	66.7 ± 10.8
*Echocardiography*	
LVEF, %*	48 (30–58)
LVEF categories, *n* (%)	
≤40%	19 (34.5)
41–49%	10 (18.2)
≥50%	26 (47.3)
PASP, mmHg*	52 (38 – 58)
TAPSE, mm*	17 (14 – 19)
*Laboratory tests*	
Hematocrit, %	38.1 ± 7.2
Serum sodium, mmol/L	139.6 ± 4.1
Serum potassium, mmol/L	4.2 ± 0.5
Urea, mg/dL*	74 (51 – 119)
Creatinine, mg/dL	1.5 ± 0.6
eGFR, mL/min/1.73 m^2^*	44.9 (29.2 – 60.2)
Urinary sodium, mmol/L	67.9 ± 30.8
Urinary potassium mmol/L	43.5 ± 18.2
NT-proBNP, pg/mL*	5218 (2856–10878)
CA125, U/mL*	54 (22–138)
*Baseline diuretic regimen*	
4 diuretics, *n* (%)	1 (1.8)
3 duretics, *n* (%)	38 (69.1)
2 diuretics, *n* (%)	15 (27.3)
1 diuretic, *n* (%)	1 (1.8)
Loop diuretics (oral), *n* (%)	55 (100)
Furosemide equivalent dose, mg*	80 (80 – 80)
Chlorthalidone, *n* (%)	46 (86.8)
Chlorthalidone, (oral) dose, mg*	12.5 (12.5–25)
MRA, *n* (%)	44 (80)
MRA, (oral) dose, mg*	25 (25–25)
Acetazolamide, *n* (%)	1 (1.8)
Acetazolamide, (oral) dose, mg	125
*Pharmacological HF therapy at baseline*	
Beta blockers, *n* (%)	51 (92.7)
ACEI/ARB, *n* (%)	25 (60)
Sacubitril-valsartan, *n* (%)	15 (27.3)
iSGLT-2, *n* (%)	13 (23.6)

*ACEI*, angiotensin-converting enzyme inhibitors; *ARB*, angiotensin II receptor blockers; *CA125*, carbohydrate antigen 125; *COPD*, chronic obstructive pulmonary disease; *DBP*, diastolic blood pressure; *eGFR*, estimated glomerular filtration rate; *iSGLT*-*2*, sodium-glucose cotransporter type 2 inhibitors; *LVEF*, left ventricular ejection fraction; *MRA*, mineralocorticoid receptor antagonists; *NT-proBNP*, amino-terminal pro-brain natriuretic peptide; *NYHA*, New York Heart Association; *PASP*, pulmonary arterial systolic pressure; *SBP*, systolic blood pressure; *TAPSE*, tricuspid annular plane systolic excursion; *VAS*, dyspnea visual analog scale

Continuous values are expressed as mean (SD) unless otherwise specified

*Value expressed as median (IQR)

### Changes in Surrogates of Congestion

**Fig. 1:**
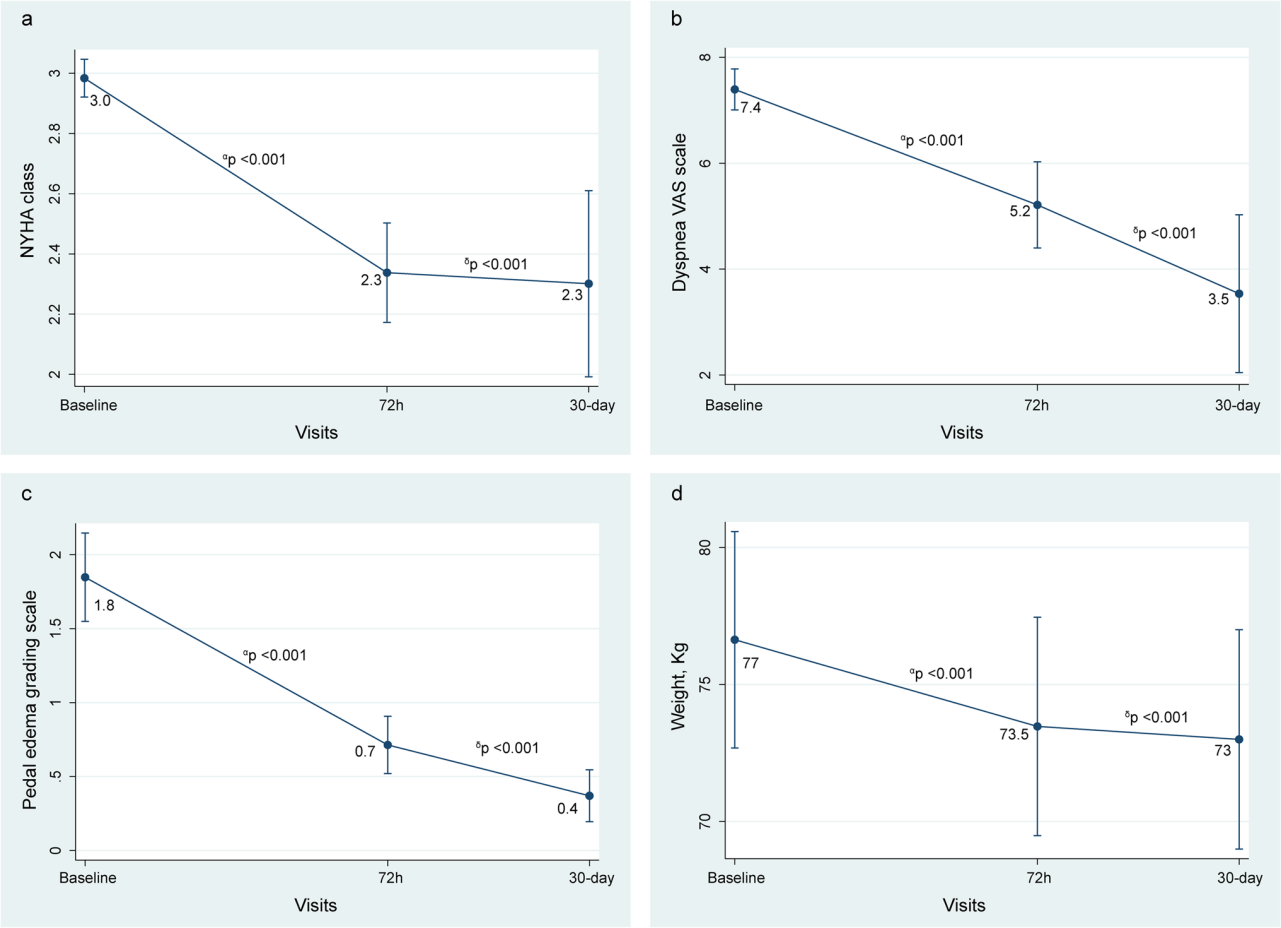
Changes in clinical surrogates of congestion. **a** NYHA class. **b** Dyspnea VAS scale. **c** Pedal edema grading scale. **d** Weight. *α,* changes at 72 h vs. baseline. *δ*, changes at FU visit vs. baseline. NYHA, New York Heart Association; VAS, visual analog scale

**Fig. 2:**
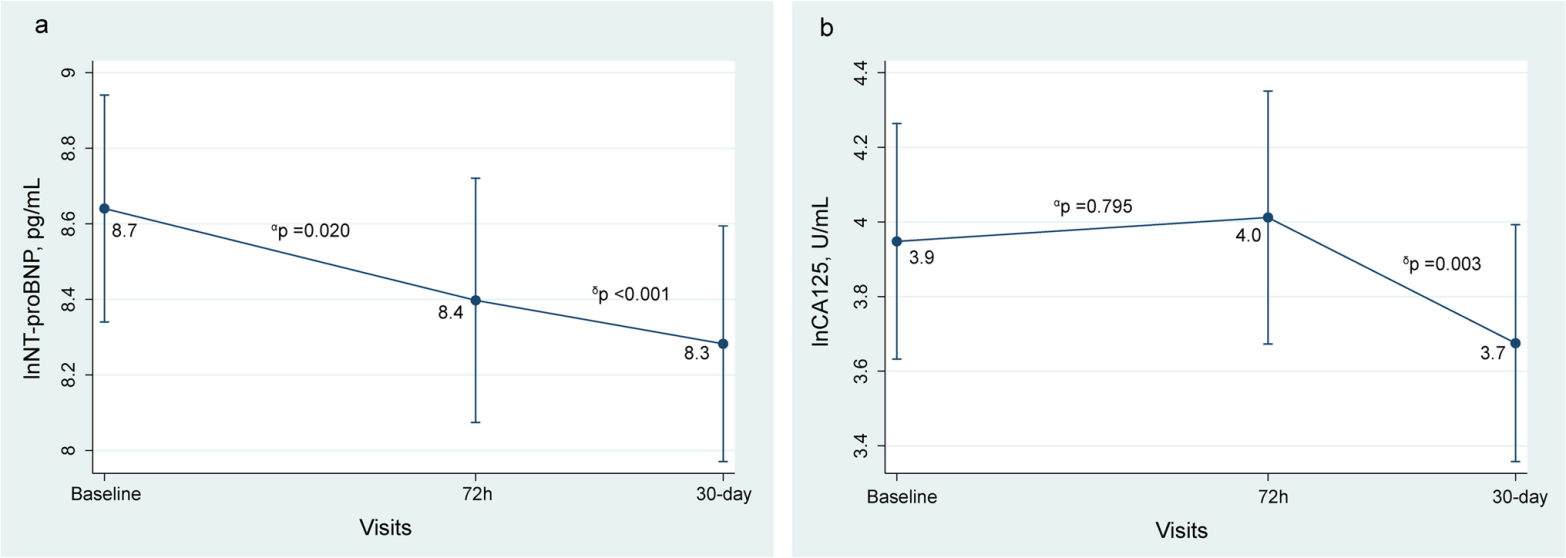
Changes in biomarkers of congestion. **a** NT-proBNP. **b** CA125. *α*, changes at 72 h vs. baseline. *δ*, changes at FU visit vs. baseline. lnCA125, natural logarithm of carbohydrate antigen 125; FU, follow-up; lnNT-proBNP, natural logarithm of amino-terminal pro-brain natriuretic peptide

Non-adjusted findings are presented in Table 2. After adjustment, we also found a significant improvement of NYHA class [72 h, Δ −0.6 (−0.9 to −0.4), *p*<0.001 and 30 days, Δ−0.7 (−1.0 to −0.3), *p*<0.001], dyspnea VAS scale [72 h, Δ −2.2 (−3.2 to −1.2, *p*<0.001) and 30 days, Δ −3.8 (−5.7 to −2.0, *p*<0.001)], pedal edema grading scale [72 h, Δ −1.1 (−1.4 to −0.9), *p*<0.001 and 30 days, Δ −1.5 (−1.8 to −1.1), *p*<0.001], and weight reduction [72 h, Δ −3.2 (−4.0 to −2.3, *p*<0.001) and 30 days, Δ −3.6 (−4.6 to −2.7, *p*<0.001)] (Fig. 1a, b, c, and d). Likewise, a significant decrease in lnNT-proBNP [72 h, Δ −0.24 (−0.45 to −0.03), *p*=0.020) and 30 days, Δ −0.35 (−0.54 to −0.18), *p*<0.001)] was ascertained (Fig. 2a). A reduction of lnCA125 was found at 30 days [Δ −0.27 −0.47 to −0.08), *p*<0.001) but not at 72 h [Δ 0.06 (0.17 to 0.30), *p*=0.795), as is shown in Fig. 2b

**Table 2:** Non-adjusted changes in efficacy and safety parameters following subcutaneous furosemide administration

Time-point	Mean change	95% CI	*p* value
Δ Urinary sodium, mmol/l			
24h	30.5	21.0 to 40.0	<0.001
48 h	29.8	20.3 to 39.3	<0.001
72 h	20.5	10.9 to 30.2	<0.001
30 days	−1.5	−12.0 to 9.1	0.788
Δ NYHA class			
72 h	−0.6	−0.8 to −0.4	<0.001
30 days	−0.8	−1.1 to −0.6	<0.001
ΔVAS			
72 h	−2.1	−3.0 to −1.2	<0.001
30 days	−3.7	−4.9 to −2.4	<0.001
Δ Pedal edema grading scale			
72 h	−1.1	−1.5 to −0.7	<0.001
30 days	−1.5	−1.9 to −1.1	<0.001
Δ Weight, kg			
72 h	−3.1	−4.0 to −2.2	<0.001
30 days	−3.7	−4.6 to −2.5	<0.001
Δ lnNTproBNP			
72 h	−0.52	−0.99 to −0.05	0.031
30 days	−0.24	−0.64 to 0.17	0.252
Δ lnCA125			
72 h	0.07	−0.14 to 0.28	0.526
30 days	−0.27	−0.44 to −0.10	0.002
Δ Systolic blood pressure, mmHg			
72 h	−7.4	−16−1 to 1.3	0.098
30 days	−13.8	−21.1 to 6.5	<0.001
Δ Estimated glomerular filtration rate, ml/min/1.73m^**2**^			
72 h	0.2	−9.1 to 9.5	0.964
30 days	−2.8	−10.1 to 5.3	0.495
Δ Serum potassium, mmo/l			
72 h	−0.29	−0.53 to −0.15	0.018
30 days	0.05	−0.15 to 0.26	0.620
Δ Serum sodium, mmo/l			
72 h	−1.1	−2.8 to 0.5	0.186
30 days	−0.9	− 2.4 to 0.5	0.208

*lnCA125*, natural logarithm of plasma antigen carbohydrate 125; *lnNT-proBNP*, natural logarithm of plasma amino-terminal pro-brain natriuretic peptide; *NYHA*, New York Heart Association; *VAS*, visual analog scale

### Changes in uNa+

**Fig. 3:**
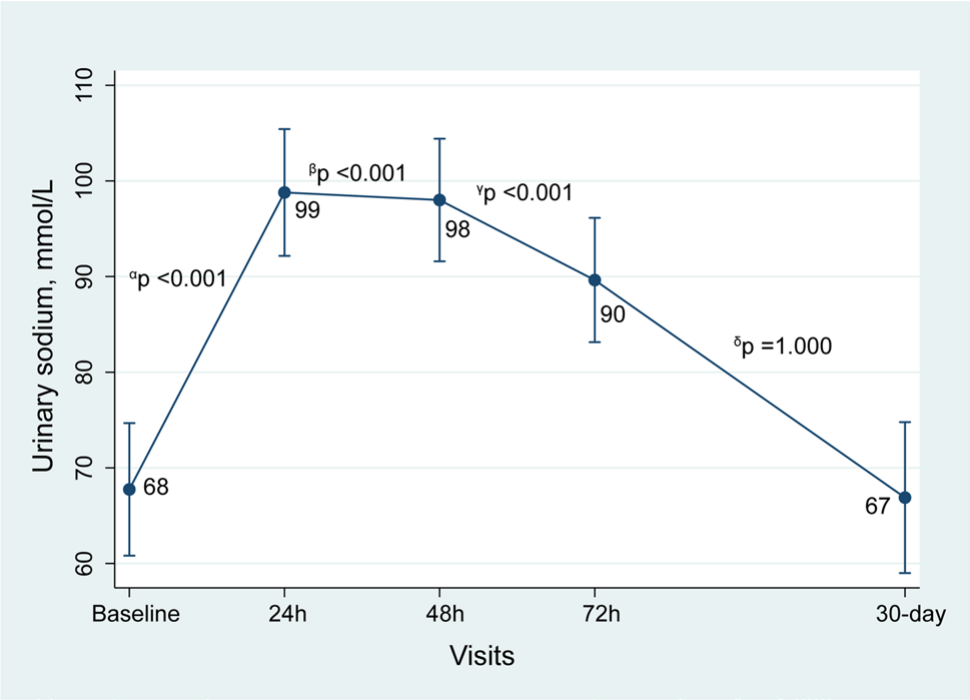
Longitudinal trajectory of uNa+ during follow-up. *α*, changes at 24 h vs. baseline. *β*, changes at 48 h vs. baseline. *γ*, changes at 72 h vs. baseline. *δ*, changes at FU visit vs. baseline. FU, follow-up; uNa+, urinary sodium

Following subcutaneous furosemide administration, uNa+ increased up to 72 h and returned to values close to baseline at 30 days (Table 2). After multivariate adjustment, we confirmed a significant and steady increase at three time-points during the first 72 h compared to baseline values (Fig. 3). LSM showed significantly higher values at 24 h [Δ31 mmol/l (22 to 40), *p*<0.001], 48h [Δ 30 mmol/l (21 to 40, *p*<0.001), and 72 h [Δ 22 mmol/l (12 to 32), *p*<0.001] compared to baseline. At 30-day follow-up, no differences were found compared to baseline [Δ −0.8 mmol/l (−13 to 11), *p*=1.00] (Fig. 3). A sensitivity analysis showed a greater natriuretic response in those patients with uNa+ <70mmol/L at baseline (*p* value for interaction <0.001) (Supplementary file 1).

### Changes in Safety Parameters

**Fig. 4:**
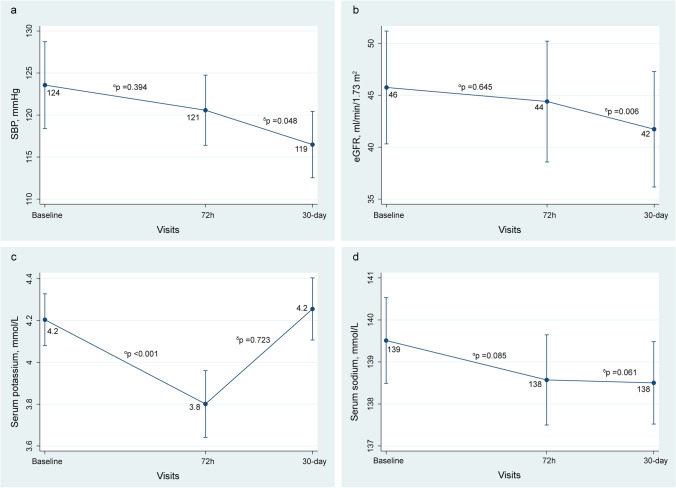
Safety parameters. **a** Systolic blood pressure. **b** Estimated glomerular filtration rate. **c** Serum potassium. **d** Serum sodium. *α*, changes at 72 h vs baseline. *δ*, changes at FU visit vs baseline. eGFR, estimated glomerular filtration rate; FU, follow-up; SBP, systolic blood pressure

Non-adjusted findings are shown in Table 2. After multivariate analyses, we found a significant drop in SBP at 72 h and 30 days (Fig. 4a) without symptomatic episodes of hypotension. Compared to baseline, eGFR did not significantly change at 72 h, but a significant decrease was observed at 30 days (Fig. 4b). Seven patients (12.7%) experienced a creatinine increase >0.5 mg/dL at 30 days. Serum potassium decreased at 72 h. No significant changes for serum sodium were found (Fig. 4c and d).

### Adverse Clinical Events

At 30 days s, we registered four deaths (three due to HF progression and one due to an ischemic cerebrovascular event) and four HF hospitalizations. A new SCF was administered in two patients due to persistent signs of congestion during the first month. In one patient, we registered cellulitis in the injection site that required a visit to the emergency room and the administration of oral antibiotics with a successful clinical evolution.

## Discussion

This retrospective analysis provides “real-world” data about the short-term efficacy and safety of SCF in the outpatient management of WHF episodes. The most relevant findings were as follows: (1) in patients presenting with WHF and volume overload, switching oral loop-diuretics to SCF (1:1.25 dose conversion) resulted in a significant and steady increase in natriuresis at 24, 48, and 72h compared to baseline values. Interestingly, this enhanced natriuretic effect was more pronounced in those with lower uNa+ (<70 mmol/L) at baseline. (2) This strategy was associated with short-term clinical evidence of decongestion (improvements in NYHA class, dyspnea VAS scale, pedal edema, and weight loss) and with statistically significant reductions in NT-proBNP (at 72h and 30 days) and CA125 (at 30 days) values. (3) SCF was overall well-tolerated, without meaningful local side effects, electrolyte disturbances, or discernible deterioration in renal function status. In summary, these preliminary data raise the possibility that this strategy may be effective for the outpatient management of WHF.

Most patients with chronic HF experience worsening signs and symptoms after a period of clinical stability that requires escalation of oral diuretic therapy. Although this strategy is commonly effective in mild cases, patients presenting with WHF and overt congestion often require parenteral diuretic administration to achieve adequate decongestion [1, 2]. However, intravenous (IV) therapy requires an IV line placement, restricting its use to in-hospital care or an outpatient clinic. Therefore, there is an increasing interest in searching for effective, value-based alternative diuretic strategies that may overcome the logistic disadvantages of IV therapy, enabling the management of WHF at home. One of these strategies is the outpatient diuretic treatment with SCF. For instance, prior authors have reported promising evidence of benefit in terms of symptoms improvement and weight loss with this diuretic strategy in small observational studies, mainly focused on palliative care [3, 4, 10, 11]. Additionally, one small mechanistic randomized study reported complete bioavailability and equivalent diuresis and natriuresis with SCF compared with similar doses of conventional IV furosemide [12]. Another small randomized phase II pilot clinical trial reported similar findings in patients with WHF [13]. Study participants were randomly assigned, in a 1:1 ratio, to receive a single dose of IV furosemide based on the subject’s outpatient oral dose (mean dose 123 ± 47 mg) or a fixed dose of 80 mg of SCF over 5h (30 mg in the first hour, followed by 12.5 mg/h for 4h) at the outpatient clinic. Those allocated to SCF achieved similar diuresis and weight change compared to IV furosemide, without any differences in adverse events [13]. However, it is important to highlight that both trials used a novel, investigational formulation of pH-neutral furosemide that is not yet approved for its use in daily clinical practice [12, 13]. The rationale for using a novel furosemide formulation is because the commercially available preparation has a pH of 8.3–9.0 and may cause irritation and discomfort upon administration. In the present study, we used non-formulated furosemide administered through a commercially available SC infusion system. By diluting the furosemide in 0.9% sodium chloride at standard concentrations (2 mg/ml in the present study), the pH of the solution becomes neutral [14, 15]. In fact, we only registered one local adverse event at the injection site that was successfully treated with oral antibiotics.

Even though we cannot establish direct comparisons with the IV route given the retrospective and observational nature of the present study, this home-based parenteral adjuvant therapy was effective in terms of decongestion/response to therapy and safe. Furthermore, to the best of our knowledge, this is the first study reporting the short-term trajectory of two well-established surrogate markers of congestion (NT-proBNP and CA125) after SCF administration in ambulatory patients with WHF. Interestingly, the observed spot uNa+ trajectory and 30-day changes in lnNT-proBNP and lnCA125 were similar to those previously reported in AHF patients successfully treated with IV loop-diuretics [16–18].

### Clinical Implications

The present study provides insightful and comprehensive data about the short-term benefits of SCF in selected patients presenting with WHF. Furthermore, several logistical advantages may facilitate its transition from clinical investigation to daily clinical practice. First, the infusion protocol does not require special furosemide formulations nor sophisticated infusion systems. Second, it offers a patient-centered alternative to inpatient treatment that can benefit patients (better quality of life–decongestion at home) and healthcare providers (increased capacity for attending other patients with WHF). Third, the cost of therapy is significantly lower than an admission. Finally, we envision that this home-based parenteral adjuvant therapy may also be useful in other clinical scenarios, such as in patients presenting to the emergency department for WHF in whom inpatient care is not strictly warranted, or during the inpatient transition from IV to oral diuretic therapy followed by an early discharge.

### Limitations

Several limitations need to be acknowledged. First, this was a single-center observational study with a small sample size. Therefore, our results should be considered exploratory and hypothesis-generating. Second, since we only included selected patients with WHF followed up in a specialized HF clinic, our findings may not apply to other clinical settings in which close monitoring is not possible. Third, we did not include a standardized protocol for the administration of other diuretics. Fourth, diuretic efficacy does not depend solely on the route of administration but also on several other complex mechanisms, which cannot be unraveled with this study design. Finally, we cannot compare the effects of this therapeutical approach vs. intravenous administration of loop diuretics.

## Conclusion

In patients with congestive WHF, ambulatory treatment with SCF resulted in successful decongestion and significant increase in natriuresis during the first 72 h. Further studies are warranted to confirm current findings and evaluate this approach’s efficacy, safety, and effectiveness compared to other most common depletive strategies.

## Supplementary Information

ESM 1 (PNG 99 kb)

High resolution image (TIF 14908 kb)
