# Supplementary figures and images for: Efficacy and Safety of Subcutaneous Infusion of Non-formulated Furosemide in Patients with Worsening Heart Failure: a Real-World Study
Source: J Cardiovasc Transl Res. 2021 Oct 12;15(3):644–52. doi: 10.1007/s12265-021-10173-1 (PMC9213343; doi:10.1007/s12265-021-10173-1)

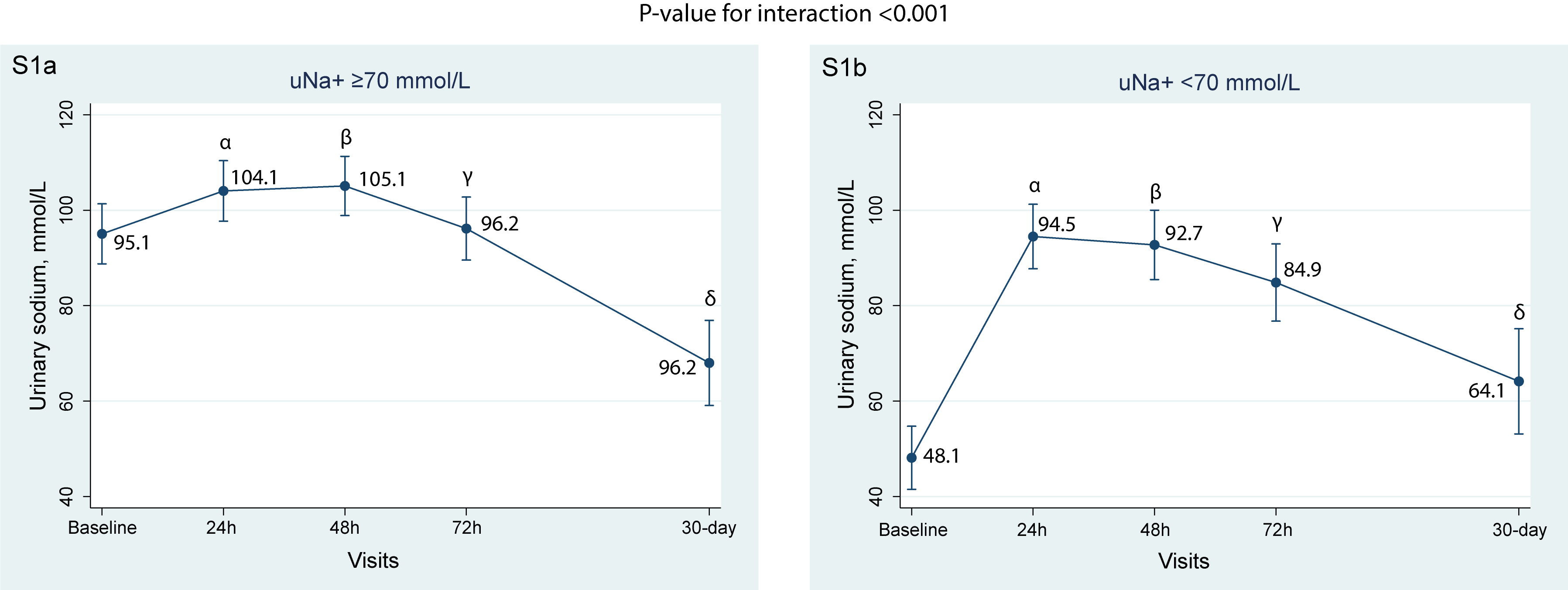

Supplement: Supplementary file 1 — (PNG 99 kb) [file 12265_2021_10173_Fig5_ESM.png]

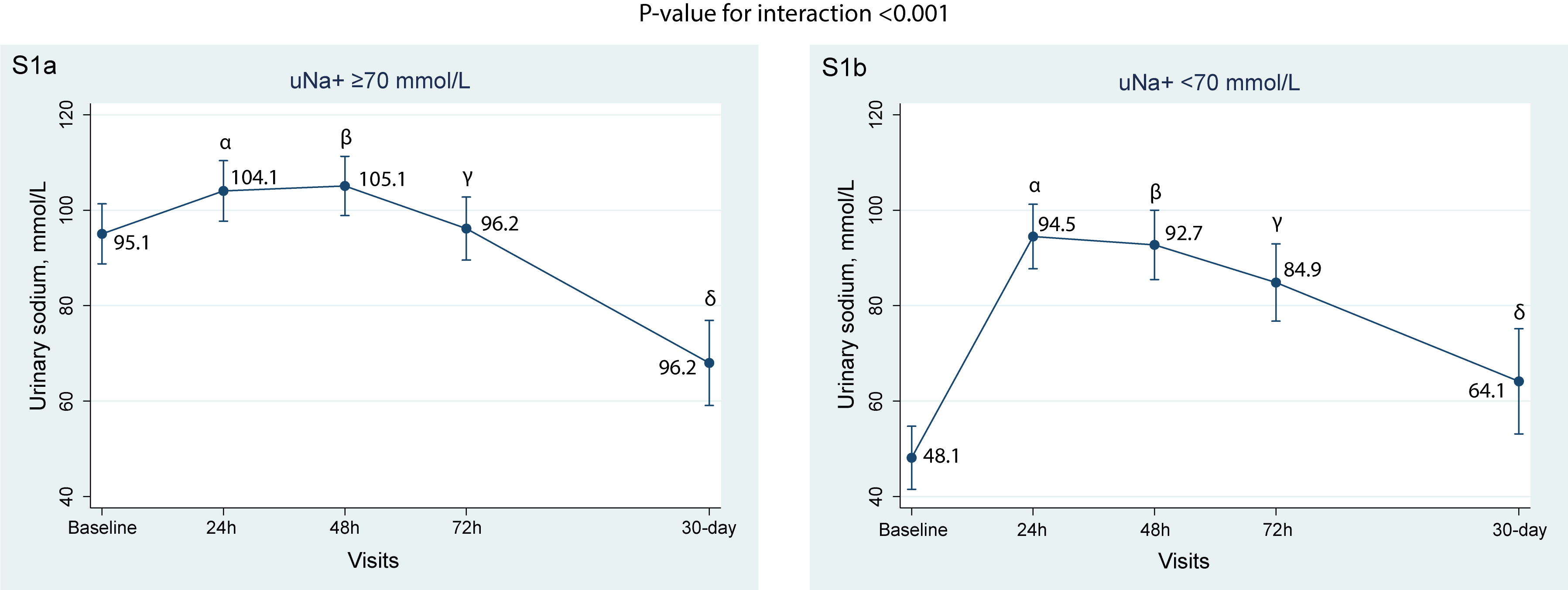

Supplement: Supplementary file 2 — High resolution image (TIF 14908 kb) [file 12265_2021_10173_MOESM1_ESM.tif]
